# Books and Magazines

**Published:** 1898-06

**Authors:** 


### Books and Magazines.

---

Principles or Guides for a Better Selection or Classification of Consumptives Amenable to High Altitude Treatment. By A. Edgar Tussey, M. D., Adjunct Professor of Diseases of the Chest in the Philadelphia Polyclinic, and Consulting Physician to the Y. M. C. A. Gymnasium, Fifteenth and Chestnut Streets, Philadelphia. Published by P. Blackiston Sons & Co., 1012 Walnut Street, Philadelphia. Price, Cloth, $1.50. 144 Pages.

This work seems to have been written as a personal remonstrance against the prevalent practice of indiscriminately recommending a change of climate to all cases of phthisis. Its literary style is somewhat unusual, but entertaining, though we cannot see that the sum of our information is increased by its publication. It is decidedly iconoclastic, and therefore would be of service to students and young practitioners by compelling them to take the sweeping generalizations of many authorities on this subject with a grain of salt.

A Treatise on Gynecology, Medical and Surgical. By S. Pozzi, M. D., member of the Academy of Medicine, Paris. Second American Edition, translated from the Third French Edition under the supervision of Brooks H. Wells, M. D., Editor American Journal of Obstetrics; Adjunct Professor of Gynecology at the New York Polyclinic; Fellow of the New York Obstetrical Society, and the New York Academy of Medicine. Six hundred illustrations. Published by Wm. Wood & Co., New York. 930 pages. Price, muslin, $5.50; leather, $6.50.

Prof. Pozzi is the most distinguished of the French gynecologists, and has contributed much to the progress of this very progressive branch of medicine.

Probably no other branch has undergone more rapid changes of practice than this one, and therefore it is absolutely necessary that those who desire to keep abreast of the times frequently read a new treatise. While the author is a Frenchman, and patriotic to the core, he has in the preparation of his work adopted the liberal motto of Voltaire, "For whoever thinks, there is neither French nor English," and he has been truly cosmopolitan in his bibliography. In our opinion, our own country has not received the credit to which it is entitled for the important work done in gynecology, but this feeling should not prevent us from availing ourselves to the fullest extent of the work of foreign surgeons. The work under review is a very valuable one, and presents many of the problems of gynecology in a new light. It is very full, for a text-book, and some of the more important subjects are especially well treated. Sixty pages are devoted to the all-important subject of "Methods of Gynecological Examination," and a like number to uterine displacements. The American editor and translators have done their tasks well, the language is faultless, and the mechanical execution is good, while the explanatory notes and remarks of the editor are of great value. Numerous and excellent illustrations are inserted throughout the book.

FLINT'S ENCYCLOPEDIA OF MEDICINE AND SURGERY. Second (1898) edition, 1555 pages, revised with the assistance of fifty-six contributors and thoroughly in line with recent advances in medical science. Cloth, $5, Leather or Half Mor., $6. Published by J. B. Flint & Co., New York. [This is not a work by the well known Professor Flint—nor by the late Professor Flint.—ED.]

---

SAMANTHA AT SARATOGA, IN A NEW DRESS. One of the funniest of all the funny books is certainly "Samantha at Saratoga." Will Carleton pronounces it "delicious humor" and Bishop Newman says it is "bitterest satire, coated with the sweetest exhiliarating fun." Formerly published by subscription at the price of $2.50, and sold, it is said, by the hundred thousand. It has recently been issued in an exquisite little cloth-bound volume in the "Cambridge Classics" series by the celebrated cheap book publishers, Hurst & Co., of New York, as a means of widely advertising that series, and is sold at the fabulously low price of 25 cents, it would seem strange if they should not sell a million of them. They are sold by booksellers, or the publishers direct.

